# Associations between cancer survivorship and subsequent respiratory disease: a systematic literature review

**DOI:** 10.1136/bmjresp-2024-002681

**Published:** 2025-06-04

**Authors:** Kirsty Andresen, Helena Carreira, Ruchika Jain, Harriet Forbes, Elizabeth Williamson, Jennifer K Quint, Krishnan Bhaskaran

**Affiliations:** 1 Non Communicable Disease Epidemiology, London School of Hygiene & Tropical Medicine Faculty of Epidemiology and Population Health, London, UK; 2 Department of Population Health, London School of Hygiene & Tropical Medicine, London, UK; 3 Medical Statistics, London School of Hygiene & Tropical Medicine Faculty of Epidemiology and Population Health, London, UK; 4 School of Public Health, Imperial College London, London, UK

**Keywords:** Respiratory Infection, Viral infection, Pneumonia, COVID-19, COPD epidemiology, Asthma

## Abstract

**Background:**

The population of cancer survivors is growing. Some cancers and their treatments may lead to long-term adverse respiratory issues. This systematic review aims to summarise the evidence on the association between cancer survivorship and long-term respiratory health, across a range of cancer types.

**Methods:**

We searched Cochrane, Embase and MEDLINE up until 23 February 2025 for cohort or nested case-control studies comparing incident respiratory outcomes in people with a history of cancer versus population-based cancer-free controls. We required studies to include follow-up time beyond the period of active cancer treatment. Outcomes included acute respiratory infections and chronic respiratory conditions. Study quality was assessed using The Scottish Intercollegiate Guidelines Network methodology checklists.

**Results:**

We identified 34 eligible cohort studies. Cancer survivors’ cohort sizes ranged from 1325 to >8 million. Only 4 out of 34 studies adjusted for smoking, leading to most studies being rated as low quality. Four of the 21 studies of acute respiratory infections were rated as acceptable/high quality, and of these, all observed raised risks, notably among survivors of haematological, head and neck, lung and oesophageal cancers. Of 19 studies of chronic respiratory conditions, 1 was rated as high quality, finding increased risks of chronic obstructive pulmonary disease (COPD) and pneumonitis in survivors of head and neck cancer. The remaining studies found increased risks of adverse outcomes from acute respiratory infections in 17 of 21 cancer types for which data were available, and of COPD in cervical, head and neck, lung, oesophageal, oral, stomach, thyroid and vulva cancers.

**Discussion:**

These findings suggest increased risks of a range of respiratory conditions in survivors of some cancers. Much of the evidence is compromised by a lack of control for key potential confounders, like smoking. Future studies should address this limitation and investigate the drivers of respiratory risks in cancer survivors. Improved evidence could inform mitigation strategies and lead to better survivorship care plans.

**PROSPERO registration number:**

CRD42022311557.

**WHAT IS ALREADY KNOWN ON THIS TOPIC**

- Some studies suggest that cancer treatments may have a long-term negative impact on the respiratory health of cancer survivors, but the evidence is not consistent. There are no previous systematic reviews of the evidence on medium to long-term respiratory risks in survivors of adult cancers.

**WHAT THIS STUDY ADDS**

- This review suggests increased risks of a range of acute and chronic respiratory conditions in survivors of some cancers. However, the current evidence base is compromised by a high risk of bias, in particular due to a lack of control for key confounders such as smoking.

**HOW THIS STUDY MIGHT AFFECT RESEARCH, PRACTICE OR POLICY**

- Our summary of the evidence helps identify key gaps, specifically, the need for well-designed studies that adjust for smoking and other key confounders. This review also highlights particular cancer survivor groups and respiratory conditions where additional research, and potentially closer clinical monitoring, may be warranted.

## Introduction

 The global population of cancer survivors was estimated to be around 43.8 million in 2018, and this number is steadily increasing.^1^ Cancer and its treatment can leave important sequelae, and survivors may experience a variety of health-related complications after the active treatment phase has ended.^2^ One particular area of interest is whether cancer survivorship is associated with long-term respiratory health.

Studies in survivors of childhood and adolescent cancers showed an association between common cancer treatments such as chemotherapy, immunotherapy, radiation and stem-cell transplantation and subsequent pulmonary toxicity^3^,^6^ that could be driven at least in part by treatment-induced impaired lung development and decreased lung function.^3 7^

However, little is known about respiratory outcomes in survivors of adult cancers. Cancer and its treatment affect the immune system, potentially leading to an increased risk of acute respiratory infections, even in the long term.^8^ Furthermore, there is concern that damage to the respiratory tract, for example, due to the toxic effects of radiotherapy and chemotherapy, could increase the risk of developing new respiratory conditions in the years after treatment. Such damage might also increase the risk of exacerbations for pre-existing chronic respiratory conditions,^9 10^ which is a key consideration given that up to a quarter of adult cancer patients have pre-existing respiratory comorbidities.^11^ On the other hand, the presence of shared risk factors between cancer and respiratory morbidities could confound associations between these two classes of disease.

We conducted a systematic review to summarise the current evidence surrounding the association between cancer survivorship and subsequent long-term respiratory outcomes in adults and assess its quality. We aimed to evaluate the relative risk of a broad spectrum of respiratory conditions across a range of cancer types, in order to build a complete picture of the evidence on respiratory health during cancer survivorship.

## Methods

This systematic review is reported in accordance with the guidance of the Preferred Reporting Items for Systematic Review and Meta-Analysis (PRISMA). The study was registered in the International Prospective Register of Systematic Reviews and followed the methods specified in the systematic review protocol,^12^ published according to the PRISMA guidance for systematic review protocols.

### Information sources and search strategy

The literature search was conducted using MEDLINE, Embase (via Ovid research platform) and Cochrane Reviews, all from database inception to 23 February 2025. The search included terms for the target population (survivors of adult cancers) and respiratory conditions of interest. These included infections such as COVID-19, influenza, pneumonia, upper-respiratory tract infections (including nasopharyngitis, rhinitis, pharyngitis, sinusitis, tonsilitis, laryngitis, tracheitis, laryngotracheitis, acute bronchitis) and chronic respiratory conditions such as asthma, chronic obstructive pulmonary disease (COPD) and interstitial lung disease (including pneumonitis and pulmonary fibrosis). The list of respiratory conditions and search terms was developed in collaboration with a respiratory consultant (JKQ). The full search expression can be found in the study protocol^12^ and in online supplemental table S1. No geographic, time or language restrictions were applied.

### Studies eligibility

We included cohort or nested case-control studies that met the following criteria: (1) included individuals diagnosed with cancer that had a follow-up time beyond the period of active treatment or the first year since cancer diagnosis; (2) included a population-based comparator cohort; (3) provided comparative measures of effect (such as risk ratios, rate ratios, HR, OR or standardised mortality ratios (SMRs)) for the incident respiratory diseases of interest or sufficient information for their calculation.

We excluded studies that did not include original data, those with duplicated data, intervention studies, cross-sectional studies, studies that did not use original data and conference abstracts. We also excluded studies where the exposed group also contained individuals without cancer (eg, families and caregivers).

### Data acquisition, screening and extraction

Studies identified from the selected databases were imported into EndNote, and duplicates were removed prior to screening. The screening was conducted in two stages. First, the titles and abstracts were checked against the selection criteria by KA. A second reviewer (RJ) independently screened a random sample of 10% of titles and abstracts to assess the validity of the initial screen. The full text of all studies included after this first screening step was then assessed independently against the selection criteria by the two researchers (KA and RJ), and conflicts were discussed until an agreement was reached.

Information on the study article (authors and publication year), population sample, study design, exposure (type of cancer), comparator and outcome definitions, follow-up, measures of effect, measures of frequency and statistical methodology was extracted by KA into a prespecified extraction spreadsheet.

We took an inclusive approach for studies that did not explicitly mention age restrictions in the inclusion criteria. For studies that focused on childhood and young adult cancer survivors, we only extracted results from age stratifications that did not include children. We included 14 different studies that used Surveillance, Epidemiology and End Results (SEER) data. Neither the cancer types nor outcomes overlapped between the studies, so these were considered independent. Where studies used overlapping data, we used the most recently published study or only reported the distinct outcomes. Missing or unclear data were extracted as ‘not reported’. If the relative effect estimate for the association between cancer survivorship and the outcome was not directly reported in the original article, we calculated an OR and 95% CIs using the measures of frequency reported within the study.

### Study quality

We assessed study quality using The Scottish Intercollegiate Guidelines Network (SIGN) cohort methodology checklists.^13^ SIGN checklists focus on aspects of study design that have been shown to have a significant effect on the risk of bias. These include evaluating the internal validity (aim), selection of subjects (comparability, recruitment, loss to follow-up, likelihood of having the outcome at study start), assessment of exposure and outcome definitions, and adjustment for confounding and statistics (ie, availability of uncertainty estimates). Within SIGN, the guidance typically limits retrospective studies to a maximum score of ‘Acceptable (+)’. For the purposes of quality assessment, we considered studies using routine electronic health records (EHRs) to be prospective (as data were collected prospectively in time, and prior to outcomes occurring); thus, we allowed EHR-based cohort studies to be rated as ‘High quality (++)’ if other criteria were met.

### Data analysis

We used text, tables and forest plots to summarise the study characteristics. The results of this review were stratified by respiratory disease, cancer type and time since diagnosis, where this information was available. To aid presentation, when results were only reported stratified by age group or stage, we combined these into a single summary effect estimate using random effects meta-analysis within each individual study. Additionally, when results were reported for various types of haematological or head and neck cancers, we consolidated them into a single estimate for haematological or head and neck cancers by meta-analysing the more detailed results within each individual study. No meta-analytic methods were applied to summarise effect measures between studies due to the variability of respiratory diseases, cancer types, survivorship definitions and follow-up times for which estimates were obtained.

### Patient and public involvement

The results of this study were disseminated to members of *the Beyond Cancer Voices* patient and public involvement group in a hybrid workshop. Participants provided insights into the acceptability of the research question and contextualised the findings, informing the discussion.

## Results

### Flowchart

**Figure 1:**
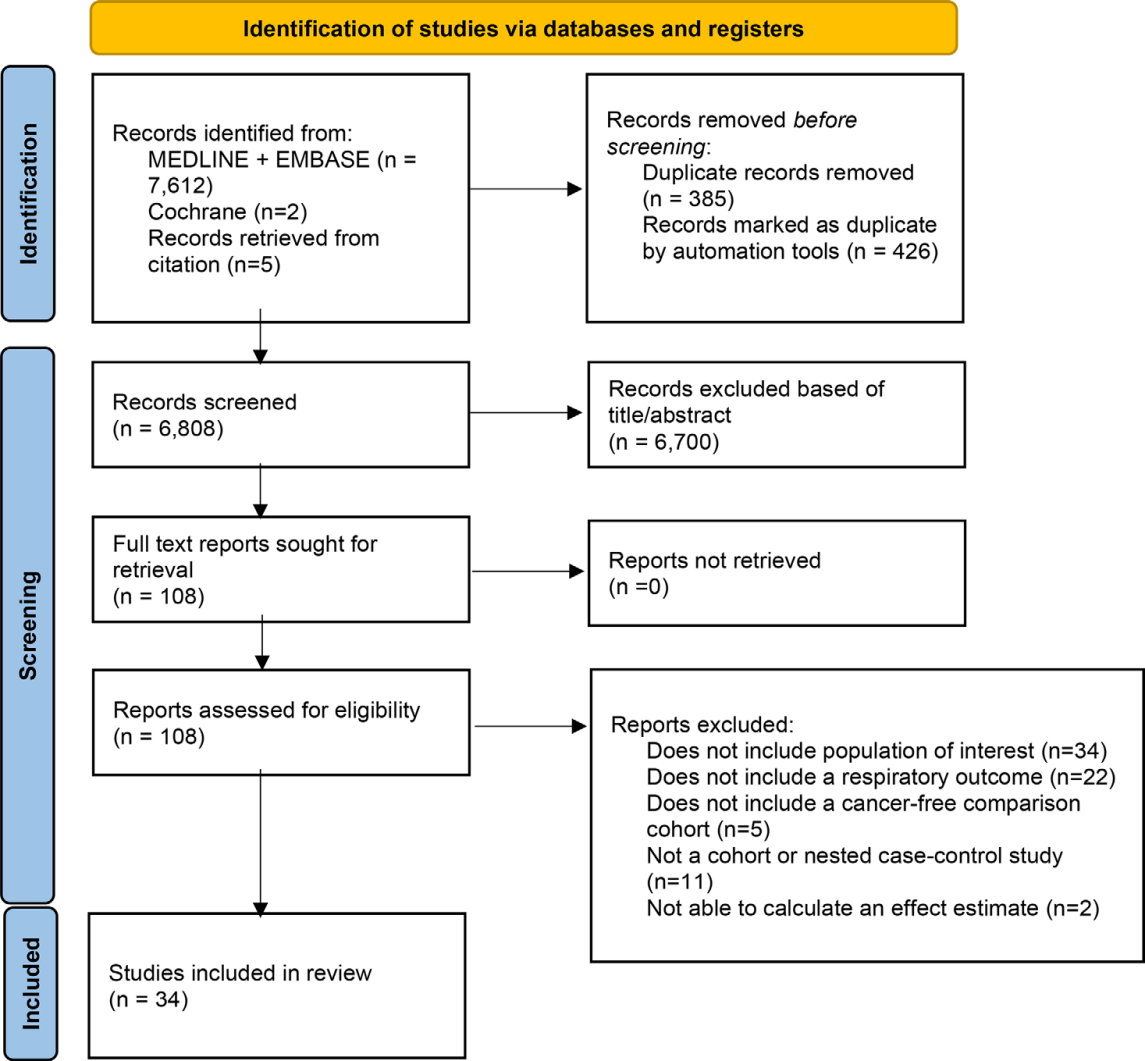
PRISMA flow diagram. Adapted from: Page *et al*.^54^ PRISMA, Preferred Reporting Items for Systematic Review and Meta-Analysis.

We identified a total of 7627, of which 108 articles were assessed for eligibility with full text review. Finally, 34 were included in the review (figure 1). Sensitivity of the title and abstract screening was 100%, and specificity was 98.7% (comparing the initial assessment by KA against the final agreed determination after reconciliation of both reviewers). The list of excluded studies is available in online supplemental table S2.

### Characteristics of included studies

Our review only included cohort studies, with no nested case-control studies meeting our criteria. See table 1 for details on the characteristics of included studies. Full results for studies including more than 15 cancer–outcome pairs are provided in online supplemental figures S1 and S2. The sample size of the cancer survivors’ cohorts ranged from 1325^14^ to over 8 million.^15^ Studies used data from cancer registries that included general population estimates (n=12), population-based administrative health records (n=7), a combination of both (n=10) and established cohort studies which could also be linked to EHRs (n=5). The most common study location was North America (n=117), with these studies commonly using SEER data (n=13). The remaining studies were conducted in Europe (n=6), Australia (n=4), the UK (n=3), Asia (n=3) and in multiple countries (n=1). While most studies reported information on wide age ranges, others focused exclusively on young adult populations (populations <40 years old, n=2) or on older adult populations (populations >40 years old, n=10).

**Table 1:** Characteristics of the included studies

Study	Setting (Data source & country)	N comparison group	N exposed group	Follow-up (max; start of follow-up)	Age group	Cancer type	Respiratory outcomes
Abalo *et al*^34^ 2024	National Patient Register (Sweden)	15 571	1559	13 years; from Dx	≥18 years	Lymphoma*	Diagnosis (acute respiratory infections†, LRTI, URTI)
Baade *et al*^25^ 2006	Queensland Cancer Registry & RGBD (AUS)	NR (general population)	144 679	20 years; from Dx	20–79 years	All cancers, colorectal, lung, melanoma	Death (any respiratory disease)
Bigelow *et al*^21^ 2020	SEER & Medicare-linked database (USA)	4994	2497	5 years; from Dx	≥66 years	Head and neck ‡	Dx (COPD, pneumonia)
Carreira *et al*^10^ 2020	CPRD GOLD & HES APC & NCRAS & IMD & ONS (ENG)	523 541	108 215	25 years; 1 year after Dx	≥18 years	All cancers, haematological, solid cancers	Hospitalisation/death (influenza)
Deckx *et al*^44^ 2012	Registration Network Family Practices (NLD)	11 973	3835	13 years; from Dx	≥60 years	All cancers	Dx (COPD)
Elmehrath *et al*^36^ 2021	SEER (USA)	NR (General population)	82 590	16 years; from Dx	NR	Ovarian	Death (acute respiratory infections†, COPD)
Fidler *et al*^41^ 2018	TYACSS and the National Death Registration System (ENG)	NR (General population)	200 945	43.2 years; 5 years after Dx	20–39	All cancers	Death (any respiratory disease, pneumonia, COPD, fibrosis, pneumonitis)
Fossa *et al*^29^ 2007	Nationwide cancer registries (DNK, SWE, NOR, FIN) & Ontario Cancer Registry (CAN) & SEER (USA)	NR (general population)	38 907	57 years; 1 year after Dx	NR	Testicular	Death (any respiratory disease, pneumonia)
Gon *et al*^43^ 2024	National Cancer Register (Japan)	NR (general population)	3 990 661	4 years; from Dx	NR	All cancers	Death (pneumonia, COPD)
Jiang *et al*^35^ 2024	SEER (USA)	NR (general population)	30 538	11 years; from Dx	NR	Oral cancer	Death (any respiratory disease, acute respiratory infections, COPD)
Jordan *et al*^16^ 2014	BOW cohort study & linked EMR (USA)	1361	1361	10 years; 5 years after Dx	65 years at Dx	Breast (early stage)	Dx (COPD)
Kawakita *et al*^22^ 2020	The Utah Population Database, Utah Cancer Registry & SEER (USA)	7796	1901	16 years; from Dx	≥18 years	Head and neck‡	Dx (any respiratory disease, acute respiratory infections, COPD, pneumonitis)
Kjaer *et al*^17^ 2019	Danish Cancer Registry & DNPR (DNK)	2 121 567	458 646	17 years; 1 year after Dx	≥40 years	Breast, colon, lung, prostate	Dx (any respiratory disease)
Lash *et al*^18^ 2014§	Six integrated healthcare systems (USA)	1361	1361	9 years; 6th yr after Dx	≥65 years	Breast¶	Dx (fibrosis, influenza, pneumonia, pneumonitis, URTI)
Lavi *et al*^30^ 2020	1991 CanCHEC & linked data (CAN)	1 300 295	1950	10 years; NR	≥25 years	Testicular	Death (any respiratory disease)
Liao *et al*^31^ 2024	SEER (USA)	NR (general population)	116 437	20 years; from Dx	NR	Stomach cancer	Death (acute respiratory infections†, COPD)
Mangone *et al*^45^ 2021	Local Health Authority of Reggio Emilia (ITA)	512 050	27 386	24 years (infection), 45 days from COVID-19 Dx (hospitalisation & death); at Dx	NR	All cancers¶	Dx, hospitalisation, death (COVID-19)
Mani *et al*^46^ 2024	SEER (USA)	NR (General population)	4 020 669	NR; from Dx	NR	All cancers	Death (COVID-19)
Ng *et al*^19^ 2018 (A)	PBS dispensing claims data (AUS)	34 610	3461	11 years; at receipt of hormone therapy	NR	Breast (hormone-dependent)	Dx (COPD)
Ng *et al*^27^ 2018 (B)	PBS dispensing claims data (AUS)	30 250	3025	6 years; at receipt of ADT	NR	Prostate	Dx (COPD)
Ording *et al*^20^ 2019	DNPR, Danish Cancer Registry & civil registration system (DNK)	162 015	32 403	14 years; 5 years after Dx	NR	Breast cancer	Dx (COPD)
Peng *et al*^32^ 2022	SEER (USA)	NR (general population)	10 179	15 years; from Dx	NR	Bladder cancer**	Death (any respiratory disease)
Prasad *et al*^39^ 2012	Finnish Cancer Registry & National Population Register Finland (FIN)	NR (general population)	9245	37 years; 5 years after Dx	20–34	All cancers	Death (any respiratory disease)
Shin *et al*^40^ 2010	Korean Central Cancer Registry & Korean National Statistical Office (KOR)	NR (general population)	243,71	12 years; at Dx	20–79	All cancers	Death (any respiratory disease, chronic respiratory disease, pneumonia)
Spoor *et al*^14^ 2023	Lifelines Cohort Study (NLD)	5300	1325	>10 yrs; from Dx	NR	Breast cancer	Dx (asthma, COPD)
Tanaka *et al*^23^ 2023	Japan Public Health Centre based prospective study (JPN)	48 947	14 520	22 years; from Dx	40–69 years	All cancers, lung, oesophageal, head and neck‡, haematological, other	Death (pneumonia)
Wang *et al*^37^ 2022	SEER (USA)	NR (general population)	183 641	41 years; from Dx	NR	Thyroid	Death (acute respiratory infections†, COPD)
Weiner *et al*^28^ 2021	SEER (USA)	NR (general population)	752 092	16 years; from Dx	NR	Prostate	Death (any respiratory disease, acute respiratory infections†)
Williamson *et al*^26^ 2020	OpenSAFELY (ENG)	16 322 018(calc)	956 374(calc)	NR	≥18 years	Haematological, non-haematological cancer	Death (COVID-19)
Xue *et al*^38^ 2023	SEER (USA)	NR (general population)	135 831	15 years; from Dx	NR	Uterine cancer ††	Death (acute respiratory infections†, COPD)
Ye *et al*^42^ 2019	Tasmanian Cancer Registry & Cause of death Unit Record File (AUS)	NR (general population)	21 637	9 years; from Dx	≥65 years	All cancers	Death (lung disease)
Yu *et al*^33^ 2022	SEER (USA)	NR (general population)	106 118	18 years; from Dx	NR	Kidney cancer ‡‡	Death (any respiratory disease, acute respiratory infections†)
Zheng *et al*^24^ 2021 (A)	SEER (USA)	NR (general population)	7 846 370	41 years; from Dx	≥40 years	All cancers, lung, head and neck‡, oesophageal, cervical, vulva	Death (COPD)
Zheng *et al*^15^ 2021 (B)	SEER (USA)	NR (general population)	8 471 051	41 years; from Dx	NR	All cancers, brain, oesophageal, haematological, lung, Kaposi sarcoma, head and neck‡, testicular, cervical, liver, bones and joints, pancreas	Death (acute respiratory infections†)

Please note that ‘NR’ stands for ‘not reported’. *p: propensity score matched. All studies included had a cohort study design.

* Lymphoma is defined as mantle cell lymphoma.

† Acute respiratory infections include pneumonia and influenza.

‡ Subsites included oral, pharynx and larynx for Zheng *et al*^24^ (A) and Zheng *et al*^15^ (B), oropharyngeal cancer only for Bigelow *et al*^21^ while subsites included oral cavity, oropharynx, hypopharynx and larynx in Kawatika *et al*,^22^ and lip, oral cavity, pharynx and larynx.

§ COPD outcomes for Lash *et al*^18^ have not been included in this study due to duplication of data with Jordan *et al*.^16^

¶ In studies reporting estimates for more than 15 cancer types/respiratory outcomes, we describe the estimates in all cancers or the most common respiratory outcome in each of the categories stated in table 1. The remaining results are presented in supplementary material figures S1 and S2online supplemental figures S1 and S2.

** Bladder cancer is defined as upper tract urothelial carcinoma.

†† Uterine cancer is defined as endometrial cancer.

‡‡ Kidney cancer is defined as renal cell carcinoma.

ADT, androgen deprivation therapy; AUS, Australia; BOW, Breast Cancer Treatment Effectiveness in Older Women; CAN, Canada; CanCHEC, The Canadian Census Health and Environment Cohorts; COPD, chronic obstructive pulmonary disease; CPRD GOLD, Clinical Practice Research Datalink GOLD; DNK, Denmark; DNPR, Danish National Patient Register; Dx, diagnosis; EMR, electronic medical records; ENG, England; FIN, Finland; HES-APC, Hospital Episode Statistics – Admitted Patient Care; JPN, Japan; KOR, Korea; LRTI, lower respiratory tract infections; NCRAS, National Cancer Registration and Analysis Service; NLD, Netherlands; NOR, Norway; ONS, Office for National Statistics; PBS, Pharmaceutical Benefits Scheme; RGBD, registrar-general births deaths and marriages; SEER, Surveillance, Epidemiology, and End Results; SWE, Sweden; TYACSS, Teenage and Young Adult Cancer Survivor Study; URTI, upper respiratory tract infections.

13 studies presented results for all types of cancer combined. Studies provided data for site-specific cancers including breast (n=6),^1416^,^20^ head and neck (n=5),^1521^,^24^ lung (n=5),^1517 23^,^25^ haematological (n=4),^10 15 23 26^ oesophageal (n=3),^15 23 24^ prostate (n=3),^17 27 28^ testicular (n=3),^15 29 30^ cervical (n=2),^15 24^ colon/colorectal (n=2),^17 25^ stomach (n=1),^15 31^ bone and joint (n=1),^32^ bladder (n=1),^15^ brain (n=1),^15^ Kaposi sarcoma (n=1),^33^ kidney (n=1),^15^ liver (n=1),^25^ lymphoma (n=1),^34^ melanoma (n=1),^25^ oral (n=1),^35^ ovary (n=1),^36^ pancreas (n=1),^15^ thyroid (n=1),^37^ uterine (n=1)^38^ and vulva (n=1).^15^

### Any respiratory disease and chronic respiratory conditions

**Figure 2:**
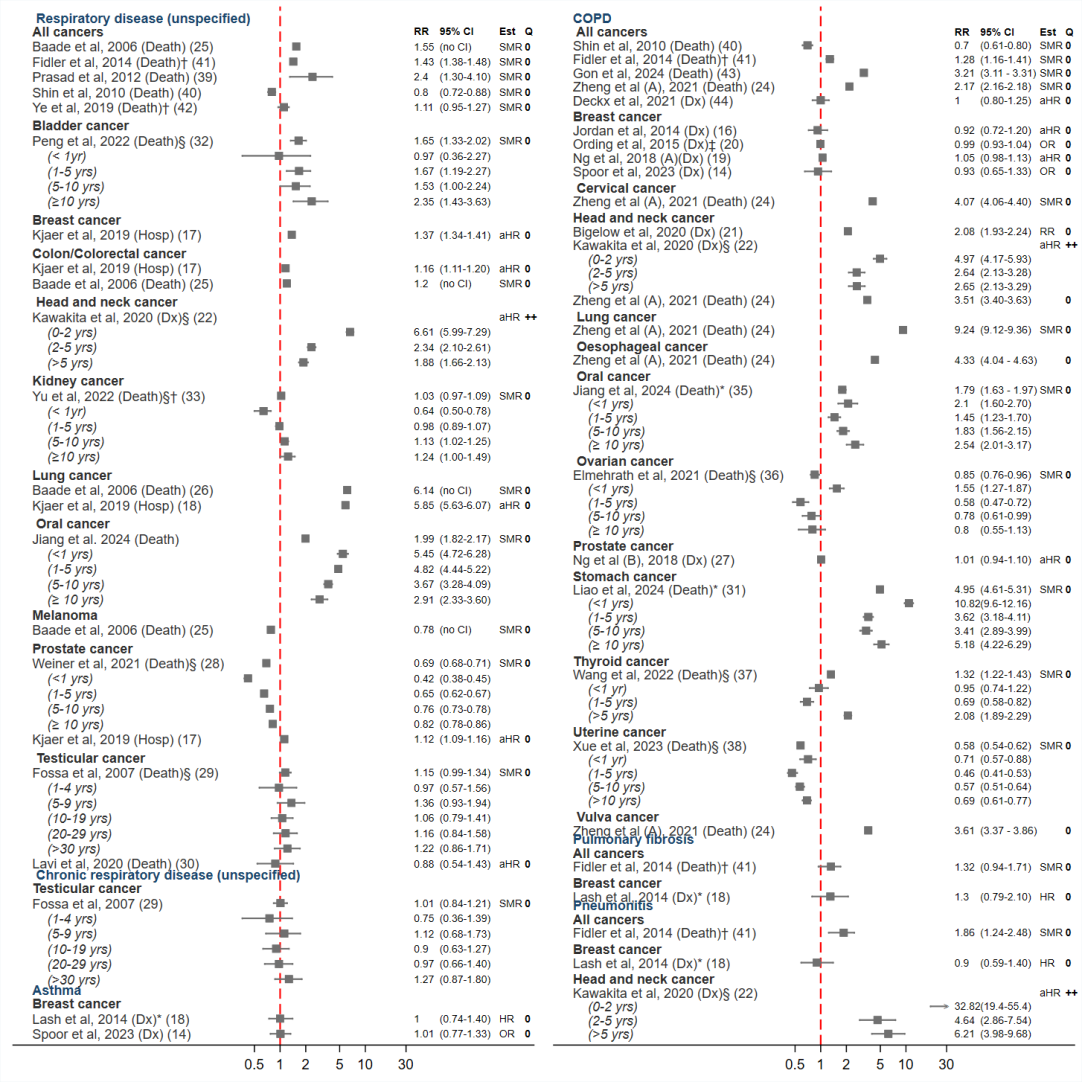
Forest plot of effect estimates (RR), 95% CIs and quality assessment scores for included studies reporting the association between cancer survivorship and chronic respiratory disease outcomes, specifically, respiratory disease (unspecified), chronic respiratory disease (unspecified), asthma, chronic obstructive pulmonary disease (COPD), pulmonary fibrosis and pneumonitis. aHR, adjusted HR; Dx, diagnosis; Est, estimate; Hosp, hospitalisation; Q, quality; RR, relative risk; SMR, standardised mortality ratio. Unless otherwise specified, the effect estimate includes all follow-up time. Quality: ‘0’=low quality; ‘+’=acceptable; ‘++’=high quality. *In studies reporting estimates for more than 15 cancer types/respiratory outcomes, we describe the estimates in all cancers or the most common respiratory outcome. The remaining results are presented in online supplemental figure S1. †Estimate calculated by meta-analysing the age-specific estimates (for groups >18 years) provided in the original study. ‡Estimate calculated from data provided in the study. §Estimates stratified by time-since diagnosis.

Figure 2 shows 13 studies compared incidence, hospitalisation or death from any respiratory disease (unspecified) in cancer survivors and in non-cancer controls. The five articles that considered all cancers combined reported conflicting results on the risk of death from respiratory conditions: three reported an increased risk in cancer survivors compared with the general population, while two reported no difference in risk or an inverse association.^2539^,^42^

A study using the linked population-based registers in Denmark showed that survivors from breast, colon, lung and prostate cancer were at increased risk of hospitalisation from respiratory diseases compared with individuals with no history of cancer.^17^ Raised risks in colorectal, colon and lung cancer were also seen in an Australian study of respiratory mortality.^25^ Studies in survivors of bladder, head and neck cancers and oral cancers also found substantially increased risks of respiratory disease,^22 32 35^ but this was not the case for kidney cancer, melanoma, prostate or testicular cancer.^25 28 29 33^

Figure 2 also shows the relative effect estimates for the 19 studies that provided data for chronic respiratory conditions.

#### Asthma

Asthma outcomes were assessed in two studies of breast cancer survivors. Both found no association between breast cancer survivorship and subsequent incident asthma.^14 18^

#### COPD

COPD outcomes were reported in 17 studies. Zheng *et al* reported that cancer survivors >40 years old in the USA had over double the risk of death from COPD than the general population (though this was not adjusted for important confounders, such as smoking).^24^ Gon *et al* also reported over a threefold risk in Japan,^43^ and three other studies combining all cancer types had mixed results.^40 41 44^ Within specific cancer types, three studies showed a pronounced risk of COPD diagnosis in head and neck cancer survivors.^21 22 24^ There was evidence of higher risks of diagnosis/death from COPD in lung, oesophageal, oral, stomach, thyroid and vulva cancer survivors but no evidence of raised risks in studies of breast, ovarian, prostate and uterine cancers.^16 18 27 31 35 36 40 41 44^

#### Pneumonitis

There was no clear association reported between incident diagnosis of fibrosis in survivors of breast cancer or young adults (any cancer) compared with population-based comparators.^18 41^ Kawakita *et al* found that the risk of pneumonitis was substantially increased in survivors of head and neck cancer compared with individuals with no history of cancer, especially in the 2 years after diagnosis (HR 32.92; 95% CI 19.44 to 55.39).^22^ In the UK-based Teenage and Young Adult Cancer Survivor Study, young adults (20–39 years old) also had more pneumonitis than the general population of the same age.^41^

### Acute respiratory infections

**Figure 3:**
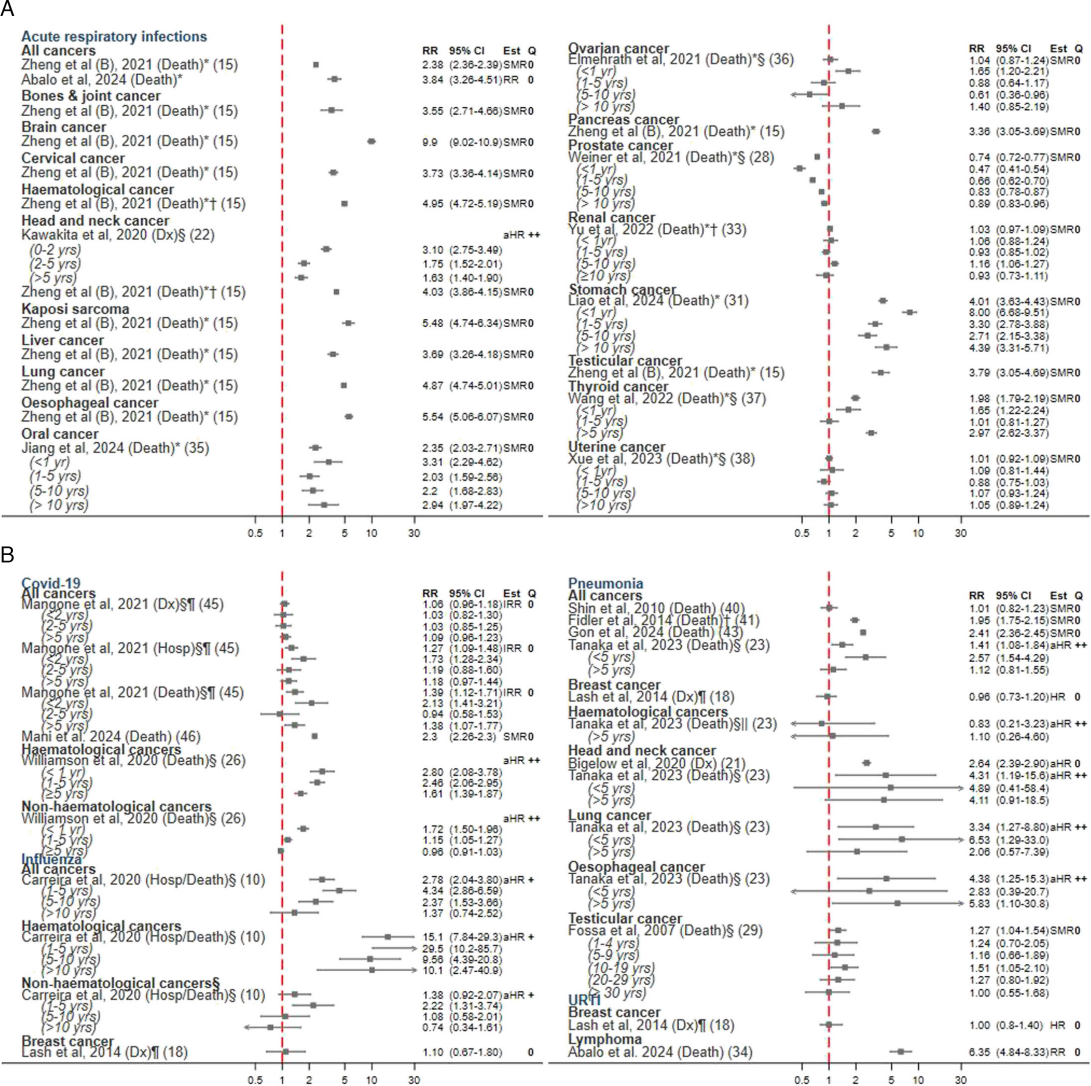
Forest plot of effect estimates (RR), 95% CI and quality assessment scores for included studies reporting the association between cancer survivorship and acute respiratory infection outcomes, specifically, acute respiratory disease (unspecified) (**A**) and COVID-19, influenza, pneumonia and upper respiratory tract infections (URTI) (**B**). aHR, adjusted HR; COPD, chronic obstructive pulmonary disorder; Dx, diagnosis; Est, estimate; Hosp, hospitalisation; Q, quality; RR, relative risk; SMR, standardised mortality ratio; URTIs, upper respiratory tract infections. Unless otherwise specified, the effect estimate includes all follow-up time. Quality: ‘0’=low quality; ‘+’=acceptable; ‘++’=high quality. *Acute infections include pneumonia and influenza. †Estimate calculated by meta-analysing the individual estimates for acute lymphatic leukaemia, acute myeloid leukaemia, Hodgkin lymphoma, myeloma and chronic myeloid leukaemia (Zheng *et al* (**B**)–haematological cancers); oral cavity, pharynx cancer and larynx cancer (Zheng *et al* (**B**)–head and neck cancers); age groups >18 years age (Fidler *et al*) and cancer stage (Yu *et al*). ^§^Estimates stratified by time since diagnosis. ^¶^In studies reporting estimates for more than 15 cancer types/ respiratory outcomes, we describe the estimates in all cancers or the most common respiratory outcomes. The remaining results are presented in the online supplemental material. ^||^Tanaka *et al* estimate stratified by <5 years since diagnosis is not reported in the original paper due to small sample size.

21 studies provided data on relative risk estimates for acute respiratory infections (figure 3). Among these, 10 used a composite outcome encompassing all acute respiratory infections (figure 3A). A US study by Kawakita *et al* focused on head and neck cancer survivors and observed an almost threefold risk of acute infections, compared with matched controls; this association was still present, though attenuated, over 5 years after cancer diagnosis.^22^ In Sweden, Abalo *et al* reported over threefold risk using hospital data linked to prescriptions.^34^ The remaining eight studies used SEER data and had pneumonia and influenza deaths as a composite measure. Zheng *et al* reported an increased SMR for acute respiratory infections in all cancers combined,^15^ as well as SMRs >3 in analyses stratified by specific types of cancer, namely for haematological, brain, oesophageal, Kaposi sarcoma, lung, head and neck, testicular, cervical, liver, bones and joints, pancreas and stomach cancers.

Other studies focused on individual cancer types. In survivors of thyroid cancer, Wang *et al* found a higher risk of death from acute respiratory infections, particularly >5 years after diagnosis (SMR 2.97; 95% CI 2.62 to 3.37).^31 33 35^ Conversely, among ovarian cancer patients, the risk was increased in the first year after cancer diagnosis (HR 1.65; 95% CI 1.2 to 2.21) only.^36^ Studies of survivors of uterine and renal cancers showed no association between cancer survivorship and respiratory infections.^33 38^ To note, Weiner *et al* reported an inverse association between prostate cancer survivorship and death due to respiratory infections.^28^ Zheng *et al* used SEER data to study over 8 million cancer survivors and observed the highest SMRs of acute infections defined as pneumonia/influenza in survivors of haematological cancers, brain cancers and head and neck cancers, Kaposi sarcoma, liver cancer, lung cancer, oesophageal cancer, pancreatic cancer and testicular cancer. Jiang *et al* and Liao *et al*—also using SEER data—found increased SMRs that persisted >10 years after diagnosis in both oral cancer and stomach cancer, respectively.^31 35^

#### COVID-19

Mangone *et al* reported regional data from Italy indicating an increased risk of COVID-19 hospitalisation and death among cancer survivors, compared with individuals in the same region with no cancer^45^ (figure 3B). The risks of hospitalisation and death were highest in the first 2 years after cancer diagnosis, particularly in haematological cancer survivors (see online supplemental figure S2). No association was observed with COVID-19 diagnosis itself.^45^ Similarly, Mani *et al* found a twofold increased risk of death in the USA, using SEER data.^46^ In a comprehensive population-based study exploring factors linked to COVID-19 mortality using UK primary care data, Williamson *et al* reported that a solid cancer diagnosis within the previous 5 years was associated with an increased risk of death due to COVID-19 (HR: 1.61; 95% CI: 1.39 to 1.87). The association was larger for survivors of haematological cancers, who had significantly elevated risks even beyond 5 years from the initial cancer diagnosis.^26^

#### Influenza

Carreira *et al* reported an increased risk of hospitalisation and/or death from influenza in survivors of all cancers combined, using UK data pre-dating the COVID-19 pandemic, but this increased risk attenuated at >10 years since diagnosis^10^ (figure 3B). Survivors of haematological cancers had a particularly elevated risk of hospitalisation and/or death from influenza, which remained 10-fold higher even more than a decade after the initial cancer diagnosis.^10^

#### Pneumonia

Eight studies provided data on pneumonia outcomes compared with population-based cohorts (figure 3B). Two studies conducted in Japan found an increased risk of pneumonia death.^23 43^ Of these, Tanaka *et al*^23^ reported HRs adjusted for various patient-level factors (including smoking) and stratified analysis by time since diagnosis. They reported an increased risk of pneumonia mortality in cancer survivors up to 5 years post-diagnosis, compared with those without a history of cancer, but relative risk estimates were no longer significantly elevated for those diagnosed at more than 5 years.^23^ Shin *et al* found similar null results in their cohort of individuals who survived >5 years.^40^ A UK study reporting data from a younger population (20–39 years) who had survived any cancer showed an increased risk of pneumonia-related mortality compared with the general population.^41^

Among studies of specific cancers, survivors of head and neck, lung and oesophageal cancers tended to have increased risks of pneumonia among all follow-up times,^21 23^ though estimates were imprecise. There was no clear association between cancer survivorship and pneumonia incidence/mortality in the studies providing data for long-term elderly breast cancer survivors (>65 years, alive >5 years after diagnosis) or testicular cancers.^18 23 29 40^ Tanaka *et al* did not find an increased risk of pneumonia death in blood cancer survivors >5 years after diagnosis, but CIs were wide.^23^

### Quality assessment

The quality assessments are presented in table 2. Three studies were scored as high quality (++) and one as acceptable (+). 30 studies were scored as low quality (0) due to key issues identified in the domains of selection of subjects and confounding.

**Table 2:** Quality of included studies according to Scottish Intercollegiate Guidelines Network (SIGN) methodology checklist for cohort studies

Quality assessment domains						Risk of bias (SIGN checklist)
Study	Aim	Selection of subjects	Assessment of exposure and outcome	Confounding	Statistical analysis	
Abalo *et al*^34^ 2024	✓	✓	✓	X (age at diagnosis, sex, calendar year, Charlson comorbidity index, year since diagnosis and a time‐dependent variable indicating the number of previous infection)	✓	Low quality (0)
Baade *et al*^25^ 2006	✓	✓	✓	X (age and sex)	X	Low quality (0)
Bigelow *et al*^21^ 2020	✓	✓	✓	X (age, race, sex, region and death)	✓	Low quality (0)
Carreira *et al*^10^ 2020	✓	X	✓	✓ (age, sex, comorbidities, smoking and deprivation)	✓	Acceptable (+)
Deckx *et al*^44^ 2012	✓	✓	✓	X (age, sex, comorbidities)	✓	Low quality (0)
Elmehrath *et al*^36^ 2021	✓	✓	✓	X (age and sex)	✓	Low quality (0)
Fidler *et al*^41^ 2018	✓	✓	✓	X (age, sex, calendar time)	✓	Low quality (0)
Fossa *et al*^29^ 2007	✓	X	✓	X (age, sex and calendar time)	✓	Low quality (0)
Gon *et al*^43^ 2024	✓	✓	✓	X (age and sex)	✓	Low quality (0)
Jiang *et al*^35^ 2024	✓	✓	✓	X (age and sex)	✓	Low quality (0)
Jordan *et al*^16^ 2014	✓	X	✓	X (age, geography, health system and existence of any prevalent comorbidity)	✓	Low quality (0)
Kawakita *et al*^22^ 2020	✓	✓	✓	✓ (age, sex, race, Charlson Comorbidity Index, race, BMI, smoking, treatment, cancer subsite and clinical disease stage)	✓	High quality (++)
Kjaer *et al*^17^ 2019	✓	✓	✓	X (age, sex, year of cancer diagnosis or study entry, income, marital status and Charlson Comorbidity Index)	✓	Low quality (0)
Lash *et al*^18^ 2014	✓	✓	✓	X (adjustment for multiple testing)	✓	Low quality (0)
Lavi *et al*^30^ 2020	✓	✓	X	X (age, marital status, education level, subcountry region of residence, urbanicity and minority status)	✓	Low quality (0)
Liao *et al*^31^ 2024	✓	✓	✓	X (age and sex)	✓	Low quality (0)
Mangone *et al*^45^ 2021	✓	✓	✓	X (age and sex)	✓	Low quality (0)
Mani *et al*^46^ 2024	✓	✓	✓	X (age and sex)	✓	Low quality (0)
Ng *et al*^19^ 2018 (breast)	✓	✓	Can’t say	X (age and comorbidities)	✓	Low quality (0)
Ng *et al*^27^ 2018 (prostate)	✓	✓	Can’t say	X (age and comorbidities)	✓	Low quality (0)
Ording *et al*^20^ 2015	✓	✓	✓	X (age and comorbidities)	✓	Low quality (0)
Peng *et al*^32^ 2022	✓	✓	✓	X (age and sex)	✓	Low quality (0)
Prasad *et al*^39^ 2012	✓	✓	✓	X (age)	✓	Low quality (0)
Shin *et al*^40^ 2010	✓	✓	✓	X (age and sex)	✓	Low quality (0)
Spoor *et al*^14^ 2023	✓	✓	Can’t say	X (age)	✓	Low quality (0)
Tanaka *et al* 2023^23^	✓	✓	✓	✓ (age, location, sex, smoking, alcohol, BMI, physical activity, caffeine intake and history of diabetes)	✓	High quality (++)
Wang *et al*^37^ 2022	✓	Can’t say	✓	X (age and sex)	✓	Low quality (0)
Weiner *et al*^28^ 2021	✓	✓	✓	X (age and sex)	✓	Low quality (0)
Williamson *et al*^26^ 2020	✓	✓	✓	✓ (age, sex, BMI, smoking, IMD quintile and comorbidities)	✓	High quality (++)
Xue *et al* 2023^38^	✓	✓	✓	X (age and sex)	✓	Low quality (0)
Ye *et al*^42^ 2019	✓	✓	✓	X (age, sex and cancer type)	✓	Low quality (0)
Yu *et al*^33^ 2022	✓	✓	✓	X (age and sex)	✓	Low quality (0)
Zheng *et al*^24^ (A), 2022	✓	✓	✓	X (age and sex)	✓	Low quality (0)
Zheng *et al*^15^ (B), 2022	✓	✓	✓	X (age and sex)	✓	Low quality (0)

Quality: ‘0’=low quality; ‘+’=acceptable; ‘++’=high quality.

BMI, body mass index; IMD, index of multiple deprivation; SIGN, Scottish Intercollegiate Guidelines Network.

In terms of confounders, the majority of data sources lacked information on smoking status, consequently, only four studies adjusted for smoking in the analysis.

The most common issues identified in the domain of selection of subjects were the lack of details on the characteristics of the general population used to compute SMR and on how ‘incident’ disease was ascertained using EHRs.

Regarding the assessment of exposure and outcome, all studies had a clear definition of the exposure and outcome. However, many studies assessed multiple outcomes without accounting for multiple testing in their analysis. An exception was the study by Lash *et al*, which conducted a Bonferroni adjustment to address multiple testing.

## Discussion

### Key findings

This systematic review identified 34 studies that provided data on the incidence or mortality of different respiratory diseases in individuals with a history of cancer compared with a population-based group. The included studies suggest a range of increased risks of acute respiratory infections among survivors of cancer, for most (but not all) cancers reported, particularly for lung cancer, oesophageal cancer, haematological cancers and cancers of the head and neck, and that persisted in the long term. For chronic respiratory conditions, the only study that adjusted for smoking found increased risks of COPD and pneumonitis in head and neck cancer survivors. Other included studies reported increased risks of COPD in cervical, head and neck, lung, oral, oesophageal, stomach, thyroid and vulva cancers. However, we found limited numbers of studies providing data for some of the respiratory conditions, as well as inconsistent information available across different types of cancer and follow-up time, making it difficult to draw firm conclusions for any of the respiratory diseases studied. The overall quality of the evidence was substantially affected by a lack of adjustment for the key potential confounder of smoking, which was only adjusted for in four studies. Other common limitations for the assessed studies were small sample sizes within each cancer type, potential bias arising from the lack of clarity on how incident disease was determined, use of composite outcomes that are difficult to interpret and lack of strategies to account for multiple testing. While the evidence is limited in quality and quantity, the higher respiratory risks observed across a range of studies are consistent with the analysis of childhood and adolescent cancers.^3^

### Strengths and limitations of this review

The strengths of this study include its replicability and transparency. The search terms for respiratory diseases were comprehensive and reviewed by a respiratory clinician (JKQ). The full protocol was developed and published in advance of conducting the review.^12^ Initial abstract screening was validated in a 10% sample, while the full-text review was duplicated in full. Study quality was assessed systematically using established criteria. Furthermore, we did not place any exclusions on the type of cancer survivors, thus covering a wide range of cancers. To the best of our knowledge, this is the first systematic review to comprehensively evaluate the risk of respiratory diseases in adult cancer survivors compared with a population-based comparator.

Our study is also subject to limitations. Though we did not exclude studies based on language, we may have missed eligible articles due to language bias, as searches were only conducted with English search terms in English language databases. This may be evidenced by the over-representation of high-income English-speaking countries within our included studies. Some studies may have been incorrectly excluded because only 10% of the title and abstract screening was duplicated; however, the potential for selection bias is very low given the 100% sensitivity found in the 10% of results double screened.

A further limitation is the lack of comparability of outcomes. Rarely were there more than two studies that assessed the same respiratory disease in the same cancer type. Even then, the type of outcome studied differed (eg, incident diagnosis vs death from respiratory disease). This coupled with the heterogeneity of the study populations included in each study precluded formal meta-analysis and hindered our ability to assess consistency of findings.

### Strengths and limitations of the evidence base

The majority of included studies aimed to quantify the general comorbidity burden or non-cancer causes of death in survivors of cancer, without a specific focus on respiratory diseases. In these types of studies where many outcomes are considered, the chances of finding spurious association increase with the number of statistical tests that are run if multiple testing is not accounted for.^47^

The limited availability of data on smoking within routinely available data was a key concern within most of the included studies. Few studies were able to adjust for smoking and other important lifestyle factors. This is particularly relevant for cancers where smoking is a major risk factor, which tended to show the strongest associations with subsequent respiratory disease. Smoking is a strong risk factor for the most common respiratory diseases and cancer.^48 49^

The study populations varied in terms of age groups, cancer survivor definition and start/length of follow-up, which challenged comparisons. Both Jordan *et al* and Lash *et al* included only breast cancer patients >65 years of age at diagnosis and >5 years from diagnosis. The American Cancer Society characterises individuals surviving >5 years as ‘long-term survivors’.^50^ However, the interpretation of such studies is not straightforward, as those most vulnerable to respiratory diseases may have the outcome within the first 5 years of survivorship and thus be excluded from the population (depletion of susceptibles), or individuals may drop out due to competing risks such as death from other causes. Furthermore, the length of follow-up and mean/median follow-up time were highly variable between studies, making estimates difficult to compare if there was no stratification by time since diagnosis, as risks may change over the survivorship journey.

In our review, many studies grouped all cancer types as a single exposure variable. The benefits of the improved power provided by increased numbers are counteracted by the loss of important granularity. Grouping cancers may mask important associations with the outcome. A similar point applies to the grouping of outcome measures: studying composite measures such as ‘any respiratory disease’ and, to a lesser extent, ‘acute respiratory infections’ may conceal important differences in associations between conditions. Studies that looked at composite measures generally found more positive associations than those that were looking at specific cancer types or specific outcomes; it is important to see what cancer types and/or outcomes are driving the increased risks within survivors of cancer.

The included studies often used general population estimates as an indirect population-based comparator to calculate SMRs. General population estimates include individuals with prior cancer, so their use as a comparator may lead to underestimation of the size of the association between cancer survivorship and outcomes.^51^

Most included studies focused on the analysis of the first outcome event during follow-up, but this may not capture the full picture of the burden of respiratory acute conditions in survivors of adult cancers. Data are limited, but it is possible that cancer survivors are more prone to exacerbations of respiratory conditions or may suffer a higher recurrence of infections compared with individuals with no history of cancer.

### Results in the context of the broader literature

Our systematic review is consistent with recent data showing that the cumulative burden of respiratory-related disease was the third most burdensome condition in cancer survivors compared with cancer-free individuals.^52^ It is also consistent with data in adult survivors of childhood cancer, who were found to have an increased incidence of both chronic respiratory conditions and respiratory infections,^7^ with respiratory conditions being the leading cause of non-cancer death in this group.^53^

### Implications for research and clinical practice

This review shows a lack of robust evidence to understand the patterns of association between cancer survivorship and subsequent long-term acute or chronic respiratory adverse effects. It is crucial to better quantify associations between cancer and respiratory outcomes and differentiate short-term from long-term effects. High-quality population-based longitudinal research is needed to quantify specific respiratory diseases in specific groups of cancer survivors, providing a clearer understanding of the association between cancer survivorship and individual respiratory outcomes. Improved reporting of population characteristics, clearer definitions of respiratory outcomes, accounting for multiple testing and adjusting for major confounders such as smoking would enhance the quality of future research in this area.

Future studies should also investigate to what extent cancer stage, cancer treatment, time since diagnosis and shared risk factors, including smoking, may drive or explain any observed association between cancer survivorship and subsequent respiratory disease. Improved evidence could inform targeting of prevention and management strategies and lead to better survivorship care and treatment plans.

### Conclusions

The findings of this review suggest increased risks of a range of respiratory conditions in survivors of certain adult cancers. Increased risks of acute respiratory infections were seen, particularly in those with a history of lung cancer, haematological cancer and cancers of the head and neck. In terms of chronic respiratory conditions, our review suggests increased risks of COPD and pneumonitis in head and neck cancer survivors, and increased risks of COPD in cervical, lung, oesophageal, oral, stomach, thyroid and vulva cancers. However, small numbers of studies for specific associations and the large potential for bias in the current evidence base demonstrate a need for further high-quality evidence in this important area of research.

## Supplementary material

10.1136/bmjresp-2024-002681 online supplemental file 1

10.1136/bmjresp-2024-002681 Uncited online supplemental file 2

10.1136/bmjresp-2024-002681 online supplemental file 3

## Data Availability

Data sharing not applicable as no datasets generated and/or analysed for this study.
