# Supplementary material for: Associations between cancer survivorship and subsequent respiratory disease: a systematic literature review
Source: BMJ Open Respir Res. 2025 Jun 4;12(1):e002681. doi: 10.1136/bmjresp-2024-002681 (PMC12142105; doi:10.1136/bmjresp-2024-002681)
Supplement: online supplemental file 3 [file bmjresp-12-1-s003.docx]

# Supplementary material

## Search terms

Table S 1. Search expression for both MEDLINE and EMBASE*

| **Search expression** |
| --- |
| 1 *Cancer Survivors/ or exp cancer survivor |
| 2 (cancer survivor* or "living with and beyond cancer" or "living with cancer").mp. |
| 3 (respiratory and (outcome* or disease* or infection* or condition* or disorder or manifestation*)).mp. |
| 4 (lung and (outcome* or disease* or infection* or condition* or disorder or manifestation*)).mp. |
| 5 (pulmonary and (outcome* or disease* or infection* or condition* or disorder or manifestation*)).mp. |
| 6 exp respiratory tract disease/ or exp lung disease/ |
| 7 ("upper respiratory infection*" or "upper respiratory tract infection*" or nasopharyngitis or rhinitis or pharyngitis or sinusitis or tonsilitis or laryngitis or tracheitis or laryngotracheitis).mp. |
| 8 respiratory tract infections/ or common cold/ or empyema, pleural/ or laryngitis/ or pharyngitis/ or rhinitis/ or sinusitis/ or whooping cough/ or exp upper respiratory tract/ or exp upper respiratory tract congestion/ or exp upper respiratory tract infection/ |
| 9 bronchitis.mp. or Bronchitis/ or Infectious bronchitis virus/ |
| 10 pulmonary disease/ or chronic obstructive/ or bronchitis, chronic/ or pulmonary emphysema/ or Bronchitis, Chronic/ or exp chronic bronchitis/ or exp bronchitis/ |
| 11 (influenza* or flu).mp. or influenza, human/ |
| 12 exp influenza/ |
| 13 Pneumonia/ or pneumonia.mp. |
| 14 exp pneumonia/ |
| 15 Asthma/ or asthma.mp. |
| 16 exp asthma/ |
| 17 (chronic obstructive pulmonary disease or COPD or chronic bronchitis or emphysema).mp. |
| 18 exp chronic obstructive lung disease/ |
| 19 interstitial lung disease.mp. or Lung Diseases, Interstitial/ |
| 20 exp interstitial lung disease/ |
| 21 Pulmonary Fibrosis/ |
| 22 (pulmonary fibrosis or lung fibrosis).mp. |
| 23 exp lung fibrosis/ |
| 24 pneumonitis.mp. |
| 25 exp coronavirus disease 2019/ or exp COVID-19/ or (Covid-19 or SARS-CoV-2 or coronavirus 19).mp. |
| 26 3 or 4 or 5 or 6 or 7 or 8 or 9 or 10 or 11 or 12 or 13 or 14 or 15 or 16 or 17 or 18 or 19 or 20 or 21 or 22 or 23 or 24 or 25 |
| 27 1 or 2 |
| 32 26 and 27 |

*Both MeSH terms and Emtree terms were included in our search expression.

Figure S1. HRs & 95% CIs of respiratory outcomes of interest in survivors of cancer compared with those without a history of cancer extracted from Lash et al.

Figure S2. Adjusted Odd Ratios (ORs) and 95% CIs of Covid-19 hospitalisations and death due to Covid-19 among survivors of cancer compared to those with no history of cancer

Table S 2. List of excluded studies

| List of excluded studies **Label** | **Title** |
| --- | --- |
| Does not include a cancer-free comparison cohort | Characteristics & outcomes of cancer patients with COVID-19: A multicentre retrospective study from India |
| Does not include a cancer-free comparison cohort | COVID-19 and Other Underlying Causes of Cancer Deaths - United States, January 2018-July 2022 |
| Does not include a cancer-free comparison cohort | Hospitalization rates and 30-day all-cause mortality among head and neck cancer patients and survivors with COVID-19 |
| Does not include a cancer-free comparison cohort | SARS-CoV-2 Testing, Positivity Rates, and Healthcare Outcomes in a Cohort of 22,481 Breast Cancer Survivors |
| Does not include a cancer-free comparison cohort | Severity and 1-month outcome of SARS-CoV-2 infection in patients with solid cancers: a Danish nationwide cohort study |
| Does not include a respiratory outcome | A population-based analysis of 30-year mortality among five-year survivors of adolescent and young adult cancer: The roles of primary cancer, subsequent malignancy, and other health conditions |
| Does not include a respiratory outcome | Age-dependent increased odds of cardiovascular risk factors in cancer survivors: Canadian Longitudinal Study on Aging cohort |
| Does not include a respiratory outcome | Burden of Long-Term Morbidity Borne by Survivors of Acute Myeloid Leukemia Treated With Blood or Marrow Transplantation: The Results of the BMT Survivor Study |
| Does not include a respiratory outcome | Causes of death and conditional survival estimates of long-term lung cancer survivors |
| Does not include a respiratory outcome | Chronic conditions and health status in older cancer survivors |
| Does not include a respiratory outcome | Chronic physical effects and health care utilization in long-term ovarian germ cell tumor survivors: A gynecologic oncology group study |
| Does not include a respiratory outcome | Cumulative burden of 144 conditions, critical care hospitalisation and premature mortality across 26 adult cancers |
| Does not include a respiratory outcome | Economic Burden of Chronic Conditions Among Survivors of Cancer in the United States |
| Does not include a respiratory outcome | For which health problems do cancer survivors visit their General Practitioner? |
| Does not include a respiratory outcome | Health Behaviors and Quality of Life of Cancer Survivors in Massachusetts, 2006: Data Use for Comprehensive Cancer Control |
| Does not include a respiratory outcome | Health status of adolescent and young adult cancer survivors |
| Does not include a respiratory outcome | Impact of Cancer History on Outcomes Among Hospitalized Patients with COVID-19 |
| Does not include a respiratory outcome | Impact of comorbidities on people with and without cancer early in the COVID-19 pandemic: An observational study |
| Does not include a respiratory outcome | Late effects in survivors of acute leukemia treated with hematopoietic cell transplantation: a report from the Bone Marrow Transplant Survivor Study |
| Does not include a respiratory outcome | Long-Term Survival and Causes of Death After Diagnoses of Common Cancers in 3 Cohorts of US Health Professionals |
| Does not include a respiratory outcome | Modifiable Risk Factors, Health Profile and Well-Being of the Elderly Diagnosed with Cancer in Italy: Passi d'Argento Surveillance System 2016-2019 Results |
| Does not include a respiratory outcome | Pulmonary and overall healthcare utilization after childhood and young adult thyroid cancer |
| Does not include a respiratory outcome | Rates of Dysphagia-Related Diagnoses in Long-Term Survivors of Head and Neck Cancers |
| Does not include a respiratory outcome | Temporal patterns of chronic disease incidence after breast cancer: a nationwide population-based cohort study |
| Does not include population of interest | Burden of multimorbidity and polypharmacy among cancer survivors: a population-based nested case-control study |
| Does not include population of interest | Causes of Death Following Endometrial Cancer Diagnosis: An Analysis of 15 Years of Follow-Up |
| Does not include population of interest | Cause-specific late mortality among 5-year survivors of childhood cancer: The childhood cancer survivor study |
| Does not include population of interest | Cause-specific long-term mortality in survivors of childhood cancer in Switzerland: A population-based study |
| Does not include population of interest | Cause-specific mortality and second cancer incidence after non-Hodgkin lymphoma: a report from the Childhood Cancer Survivor Study |
| Does not include population of interest | Chronic Comorbidities Among Survivors of Adolescent and Young Adult Cancer |
| Does not include population of interest | Chronic disease burden among cancer survivors in the California Behavioral Risk Factor Surveillance System, 2009-2010 |
| Does not include population of interest | Clinically ascertained health outcomes, quality of life, and social attainment among adult survivors of neuroblastoma: A report from the St. Jude Lifetime Cohort |
| Does not include population of interest | Distribution of hospital care among pediatric and young adult Hodgkin lymphoma survivors-A population-based cohort study from Sweden and Denmark |
| Does not include population of interest | Early Posttherapy Hospitalizations Among Survivors of Childhood Leukemia and Lymphoma |
| Does not include population of interest | Effects of cancer history and comorbid conditions on mortality and healthcare use among older cancer survivors |
| Does not include population of interest | Excess morbidity and mortality among survivors of childhood acute lymphoblastic leukaemia: 25 years of follow-up from the United Kingdom Childhood Cancer Study (UKCCS) population-based matched cohort |
| Does not include population of interest | Infections among long-term survivors of childhood and adolescent cancer: A report from the Childhood Cancer Survivor Study |
| Does not include population of interest | Late mortality among 5-year survivors of childhood cancer: a summary from the Childhood Cancer Survivor Study |
| Does not include population of interest | Late mortality and causes of death among 5-year survivors of childhood cancer diagnosed in the period 1960-1999 and registered in the Italian Off-Therapy Registry |
| Does not include population of interest | Late mortality experience in five-year survivors of childhood and adolescent cancer: The Childhood Cancer Survivor Study |
| Does not include population of interest | Late Mortality, Subsequent Malignant Neoplasms and Hospitalisations in Long-Term Survivors of Adolescent and Young Adult Haematological Cancers |
| Does not include population of interest | Long-term inpatient disease burden in the Adult Life after Childhood Cancer in Scandinavia (ALiCCS) study: A cohort study of 21,297 childhood cancer survivors |
| Does not include population of interest | Medical conditions and physical function deficits among multiple primary cancer survivors |
| Does not include population of interest | Mortality among 5-year survivors of cancer diagnosed during childhood or adolescence in British Columbia, Canada |
| Does not include population of interest | Patient-reported outcomes in survivors of childhood hematologic malignancies with hematopoietic stem cell transplant |
| Does not include population of interest | Physical and mental health status of older long-term cancer survivors |
| Does not include population of interest | Pulmonary complications in survivors of childhood and adolescent cancer. A report from the Childhood Cancer Survivor Study |
| Does not include population of interest | Pulmonary outcomes in survivors of childhood central nervous system malignancies: a report from the Childhood Cancer Survivor Study |
| Does not include population of interest | Respiratory emergency department use from diagnosis through survivorship in children, adolescents, and young adults with cancer |
| Does not include population of interest | Respiratory morbidity in young people surviving cancer: Population-based study of hospital admissions, treatment-related risk factors and subsequent mortality |
| Does not include population of interest | Risk and impact of pulmonary complications in survivors of childhood cancer: A report from the Childhood Cancer Survivor Study (CCSS). |
| Does not include population of interest | Risk of COVID-19 Infections and of Severe Complications Among Survivors of Childhood, Adolescent, and Young Adult Cancer: A Population-Based Study in Ontario, Canada |
| Does not include population of interest | The cumulative burden of surviving childhood cancer: an initial report from the St Jude Lifetime Cohort Study (SJLIFE) |
| Does not include population of interest | Risk and impact of pulmonary complications in survivors of childhood cancer: A report from the Childhood Cancer Survivor Study (CCSS) |
| Does not include population of interest | Feasibility and safety of Heavy Lifting Strength Training in Head and Neck Cancer survivors post-surgical neck dissection (the LIFTING trial) |
| Not a cohort or nested case-control study | A retrospective study of the morbidities suffered by survivors of pediatric cancer therapy |
| Not a cohort or nested case-control study | Changes in the prevalence of comorbidity in the Australian population with cancer, 2007-2014 |
| Not a cohort or nested case-control study | Chronic comorbid conditions among adult cancer survivors in the United States: Results from the National Health Interview Survey, 2002-2018 |
| Not a cohort or nested case-control study | COVID-19 in cancer patients |
| Not a cohort or nested case-control study | Prevalence and healthcare utilization of acute respiratory infections among cancer survivors in the United States: a population-based study |
| Not a cohort or nested case-control study | Prevalence of Underlying Medical Conditions Associated With Severe COVID-19 Illness in Adult Cancer Survivors in the United States |
| Not a cohort or nested case-control study | Pulmonary obstruction changes in childhood cancer survivors - treatment complications or asthma? |
| Not a cohort or nested case-control study | Sex differences in comorbid conditions, health behaviors, health care utilization, and health-related quality of life among young adult cancer survivors |
| Not a cohort or nested case-control study | The first year of the COVID-19 pandemic and health among cancer survivors in the United States |
| Not a cohort or nested case-control study | American Society of Clinical Oncology clinical evidence review on the ongoing care of adult cancer survivors: cardiac and pulmonary late effects |
| Not a cohort or nested case-control study | Late effects in survivors of teenage and young adult cancer: does age matter? |
| Not able to calculate an effect estimate | Adverse health effects after breast cancer up to 14 years after diagnosis |
| Not able to calculate an effect estimate | Adverse health effects after breast cancer up to 14 years after diagnosis |

| Does not include a cancer-free comparison cohort | Characteristics & outcomes of cancer patients with COVID-19: A multicentre retrospective study from India |
| --- | --- |
| Does not include a cancer-free comparison cohort | COVID-19 and Other Underlying Causes of Cancer Deaths - United States, January 2018-July 2022 |
| Does not include a cancer-free comparison cohort | Hospitalization rates and 30-day all-cause mortality among head and neck cancer patients and survivors with COVID-19 |
| Does not include a cancer-free comparison cohort | SARS-CoV-2 Testing, Positivity Rates, and Healthcare Outcomes in a Cohort of 22,481 Breast Cancer Survivors |
| Does not include a cancer-free comparison cohort | Severity and 1-month outcome of SARS-CoV-2 infection in patients with solid cancers: a Danish nationwide cohort study |
| Does not include a respiratory outcome | A population-based analysis of 30-year mortality among five-year survivors of adolescent and young adult cancer: The roles of primary cancer, subsequent malignancy, and other health conditions |
| Does not include a respiratory outcome | Age-dependent increased odds of cardiovascular risk factors in cancer survivors: Canadian Longitudinal Study on Aging cohort |
| Does not include a respiratory outcome | Burden of Long-Term Morbidity Borne by Survivors of Acute Myeloid Leukemia Treated With Blood or Marrow Transplantation: The Results of the BMT Survivor Study |
| Does not include a respiratory outcome | Causes of death and conditional survival estimates of long-term lung cancer survivors |
| Does not include a respiratory outcome | Chronic conditions and health status in older cancer survivors |
| Does not include a respiratory outcome | Chronic physical effects and health care utilization in long-term ovarian germ cell tumor survivors: A gynecologic oncology group study |
| Does not include a respiratory outcome | Cumulative burden of 144 conditions, critical care hospitalisation and premature mortality across 26 adult cancers |
| Does not include a respiratory outcome | Economic Burden of Chronic Conditions Among Survivors of Cancer in the United States |
| Does not include a respiratory outcome | For which health problems do cancer survivors visit their General Practitioner? |
| Does not include a respiratory outcome | Health Behaviors and Quality of Life of Cancer Survivors in Massachusetts, 2006: Data Use for Comprehensive Cancer Control |
| Does not include a respiratory outcome | Health status of adolescent and young adult cancer survivors |
| Does not include a respiratory outcome | Impact of Cancer History on Outcomes Among Hospitalized Patients with COVID-19 |
| Does not include a respiratory outcome | Impact of comorbidities on people with and without cancer early in the COVID-19 pandemic: An observational study |
| Does not include a respiratory outcome | Late effects in survivors of acute leukemia treated with hematopoietic cell transplantation: a report from the Bone Marrow Transplant Survivor Study |
| Does not include a respiratory outcome | Long-Term Survival and Causes of Death After Diagnoses of Common Cancers in 3 Cohorts of US Health Professionals |
| Does not include a respiratory outcome | Modifiable Risk Factors, Health Profile and Well-Being of the Elderly Diagnosed with Cancer in Italy: Passi d'Argento Surveillance System 2016-2019 Results |
| Does not include a respiratory outcome | Pulmonary and overall healthcare utilization after childhood and young adult thyroid cancer |
| Does not include a respiratory outcome | Rates of Dysphagia-Related Diagnoses in Long-Term Survivors of Head and Neck Cancers |
| Does not include a respiratory outcome | Temporal patterns of chronic disease incidence after breast cancer: a nationwide population-based cohort study |
| Does not include population of interest | Burden of multimorbidity and polypharmacy among cancer survivors: a population-based nested case-control study |
| Does not include population of interest | Causes of Death Following Endometrial Cancer Diagnosis: An Analysis of 15 Years of Follow-Up |
| Does not include population of interest | Cause-specific late mortality among 5-year survivors of childhood cancer: The childhood cancer survivor study |
| Does not include population of interest | Cause-specific long-term mortality in survivors of childhood cancer in Switzerland: A population-based study |
| Does not include population of interest | Cause-specific mortality and second cancer incidence after non-Hodgkin lymphoma: a report from the Childhood Cancer Survivor Study |
| Does not include population of interest | Chronic Comorbidities Among Survivors of Adolescent and Young Adult Cancer |
| Does not include population of interest | Chronic disease burden among cancer survivors in the California Behavioral Risk Factor Surveillance System, 2009-2010 |
| Does not include population of interest | Clinically ascertained health outcomes, quality of life, and social attainment among adult survivors of neuroblastoma: A report from the St. Jude Lifetime Cohort |
| Does not include population of interest | Distribution of hospital care among pediatric and young adult Hodgkin lymphoma survivors-A population-based cohort study from Sweden and Denmark |
| Does not include population of interest | Early Posttherapy Hospitalizations Among Survivors of Childhood Leukemia and Lymphoma |
| Does not include population of interest | Effects of cancer history and comorbid conditions on mortality and healthcare use among older cancer survivors |
| Does not include population of interest | Excess morbidity and mortality among survivors of childhood acute lymphoblastic leukaemia: 25 years of follow-up from the United Kingdom Childhood Cancer Study (UKCCS) population-based matched cohort |
| Does not include population of interest | Infections among long-term survivors of childhood and adolescent cancer: A report from the Childhood Cancer Survivor Study |
| Does not include population of interest | Late mortality among 5-year survivors of childhood cancer: a summary from the Childhood Cancer Survivor Study |
| Does not include population of interest | Late mortality and causes of death among 5-year survivors of childhood cancer diagnosed in the period 1960-1999 and registered in the Italian Off-Therapy Registry |
| Does not include population of interest | Late mortality experience in five-year survivors of childhood and adolescent cancer: The Childhood Cancer Survivor Study |
| Does not include population of interest | Late Mortality, Subsequent Malignant Neoplasms and Hospitalisations in Long-Term Survivors of Adolescent and Young Adult Hematological Cancers |
| Does not include population of interest | Long-term inpatient disease burden in the Adult Life after Childhood Cancer in Scandinavia (ALiCCS) study: A cohort study of 21,297 childhood cancer survivors |
| Does not include population of interest | Medical conditions and physical function deficits among multiple primary cancer survivors |
| Does not include population of interest | Mortality among 5-year survivors of cancer diagnosed during childhood or adolescence in British Columbia, Canada |
| Does not include population of interest | Patient-reported outcomes in survivors of childhood hematologic malignancies with hematopoietic stem cell transplant |
| Does not include population of interest | Physical and mental health status of older long-term cancer survivors |
| Does not include population of interest | Pulmonary complications in survivors of childhood and adolescent cancer. A report from the Childhood Cancer Survivor Study |
| Does not include population of interest | Pulmonary outcomes in survivors of childhood central nervous system malignancies: a report from the Childhood Cancer Survivor Study |
| Does not include population of interest | Respiratory emergency department use from diagnosis through survivorship in children, adolescents, and young adults with cancer |
| Does not include population of interest | Respiratory morbidity in young people surviving cancer: Population-based study of hospital admissions, treatment-related risk factors and subsequent mortality |
| Does not include population of interest | Risk and impact of pulmonary complications in survivors of childhood cancer: A report from the Childhood Cancer Survivor Study (CCSS). |
| Does not include population of interest | Risk of COVID-19 Infections and of Severe Complications Among Survivors of Childhood, Adolescent, and Young Adult Cancer: A Population-Based Study in Ontario, Canada |
| Does not include population of interest | The cumulative burden of surviving childhood cancer: an initial report from the St Jude Lifetime Cohort Study (SJLIFE) |
| Does not include population of interest | Risk and impact of pulmonary complications in survivors of childhood cancer: A report from the Childhood Cancer Survivor Study (CCSS) |
| Does not include population of interest | Feasibility and safety of Heavy Lifting Strength Training in Head and Neck Cancer survivors post-surgical neck dissection (the LIFTING trial) |
| Not a cohort or nested case-control study | A retrospective study of the morbidities suffered by survivors of pediatric cancer therapy |
| Not a cohort or nested case-control study | Changes in the prevalence of comorbidity in the Australian population with cancer, 2007-2014 |
| Not a cohort or nested case-control study | Chronic comorbid conditions among adult cancer survivors in the United States: Results from the National Health Interview Survey, 2002-2018 |
| Not a cohort or nested case-control study | COVID-19 in cancer patients |
| Not a cohort or nested case-control study | Prevalence and healthcare utilization of acute respiratory infections among cancer survivors in the United States: a population-based study |
| Not a cohort or nested case-control study | Prevalence of Underlying Medical Conditions Associated With Severe COVID-19 Illness in Adult Cancer Survivors in the United States |
| Not a cohort or nested case-control study | Pulmonary obstruction changes in childhood cancer survivors - treatment complications or asthma? |
| Not a cohort or nested case-control study | Sex differences in comorbid conditions, health behaviors, health care utilization, and health-related quality of life among young adult cancer survivors |
| Not a cohort or nested case-control study | The first year of the COVID-19 pandemic and health among cancer survivors in the United States |
| Not a cohort or nested case-control study | American Society of Clinical Oncology clinical evidence review on the ongoing care of adult cancer survivors: cardiac and pulmonary late effects |
| Not a cohort or nested case-control study | Late effects in survivors of teenage and young adult cancer: does age matter? |
| Not able to calculate an effect estimate | Adverse health effects after breast cancer up to 14 years after diagnosis |
| Not able to calculate an effect estimate | Adverse health effects after breast cancer up to 14 years after diagnosis |
| Studies included in review | A comparison of cardiovascular and pulmonary morbidities and risk factors in breast cancer survivors compared to an age-matched female control group in the Lifelines prospective population cohort |
| Studies included in review | Adverse respiratory outcomes among head and neck cancer survivors in the Utah Cancer Survivors Study |
| Studies included in review | Burden of comorbidities is higher among elderly survivors of oropharyngeal cancer compared with controls |
| Studies included in review | Cardiovascular and other competing causes of death among patients with cancer from 2006 to 2015: An Australian population-based study |
| Studies included in review | Cause of Death Among Patients With Thyroid Cancer: A Population-Based Study |
| Studies included in review | Cause of death during prostate cancer survivorship: A contemporary, US population-based analysis |
| Studies included in review | Cause of Death During Renal Cell Carcinoma Survivorship: A Contemporary, Population-Based Analysis |
| Studies included in review | Cause of death during upper tract urothelial carcinoma survivorship: A contemporary, population-based analysis |
| Studies included in review | Causes of death in endometrial cancer survivors: A Surveillance, Epidemiology, and End Result–based analysis |
| Studies included in review | Chronic Diseases among Older Cancer Survivors |
| Studies included in review | Comorbidities in Australian women with hormone-dependent breast cancer: A population-based analysis |
| Studies included in review | Comprehensive evaluation of the incidence of late effects in 5-year survivors of breast cancer |
| Studies included in review | Cumulative COVID-19 incidence, mortality and prognosis in cancer survivors: A population-based study in Reggio Emilia, Northern Italy |
| Studies included in review | Deaths from COPD in patients with cancer: a population-based study |
| Studies included in review | Development of comorbidities in men with prostate cancer treated with androgen deprivation therapy: an Australian population-based cohort study |
| Studies included in review | Factors associated with COVID-19-related death using OpenSAFELY |
| Studies included in review | Fatal Infections Among Cancer Patients: A Population-Based Study in the United States |
| Studies included in review | Incident comorbidities and all-cause mortality among 5-year survivors of Stage I and II breast cancer diagnosed at age 65 or older: a prospective-matched cohort study |
| Studies included in review | Increased risk of death from pneumonia among cancer survivors: A propensity score-matched cohort analysis |
| Studies included in review | Long-term non-cancer mortality in pediatric and young adult cancer survivors in Finland |
| Studies included in review | Long-term Somatic Disease Risk in Adult Danish Cancer Survivors |
| Studies included in review | Long-term Testis Cancer Survivors in Canada-Mortality Risks in a Large Population-based Cohort |
| Studies included in review | Noncancer causes of death in survivors of testicular cancer |
| Studies included in review | Non-cancer death causes after ovarian cancer diagnosis: A population-based cohort |
| Studies included in review | Non-cancer mortality among long-term survivors of adult cancer in Korea: national cancer registry study |
| Studies included in review | Non-cancer mortality among people diagnosed with cancer (Australia) |
| Studies included in review | Prevalence of COVID-19-related risk factors and risk of severe influenza outcomes in cancer survivors: A matched cohort study using linked English electronic health records data |
| Studies included in review | Relative mortality rates from incident chronic diseases among breast cancer survivors - A 14 year follow-up of five-year survivors diagnosed in Denmark between 1994 and 2007 |
| Studies included in review | Respiratory mortality of childhood, adolescent and young adult cancer survivors |
